# Transfection Studies with Colloidal Systems Containing Highly Purified Bipolar Tetraether Lipids from* Sulfolobus acidocaldarius*

**DOI:** 10.1155/2017/8047149

**Published:** 2017-01-23

**Authors:** Konrad H. Engelhardt, Shashank Reddy Pinnapireddy, Elias Baghdan, Jarmila Jedelská, Udo Bakowsky

**Affiliations:** Department of Pharmaceutics and Biopharmaceutics, University of Marburg, Marburg, Germany

## Abstract

Lipid vectors are commonly used to facilitate the transfer of nucleic acids into mammalian cells. In this study, two fractions of tetraether lipids from the archaea* Sulfolobus acidocaldarius *were extracted and purified using different methods. The purified lipid fractions polar lipid fraction E (PLFE) and hydrolysed glycerol-dialkyl-nonitol tetraether (hGDNT) differ in their structures, charge, size, and miscibility from conventional lipids. Liposomes were prepared by mixing tetraether lipids with cholesterol (CH) and 1,2-dioleoyl-3-trimethylammonium-propane (DOTAP) resulting in stable vectors for gene delivery. Lipoplexes were prepared by complexation of liposomes with a luciferase expressing plasmid (pCMV-luc) at certain nitrogen-to-phosphorus (N/P) ratios and optimised for the transient transfection of ovarian adenocarcinoma cells (SK-OV-3). Complexation efficacy was investigated by gel-red fluorescence assay. Biophysical properties, like size, surface charge, and morphology, were investigated by differential light scattering (DLS), atomic force microscopy (AFM), and scanning electron microscopy (Cryo-SEM), respectively, revealing structural differences between liposomes and lipoplexes. A range of stable transfecting agents containing tetraether lipids were obtained by incorporating 5 mol% of tetraether lipids. Lipoplexes showed a decrease in free gel-red with increasing N/P ratios indicating efficient incorporation of plasmid DNA (pDNA) and remarkable stability. Transfection experiments of the lipoplexes revealed successful and superior transfection of SK-OV-3 cell line compared to the commercially available DOTAP and branched polyethyleneimine (25 kDa bPEI).

## 1. Introduction

At present, transfection vectors can be broadly classified into viral and nonviral. Since early days of gene therapy, nonpathogenic attenuated viruses have been the most common delivery systems used. Currently, viral vectors are employed in over 65% of gene therapy clinical trials worldwide, with adenoviruses being the most common, followed by retroviruses. Viruses possess an innate ability to insert their own genetic material into the host cell; therefore, high transfection efficiency is usually observed in a variety of human tissues. Despite this significant advantage, there are several concerns over the safety of viral vectors. These include the potential to induce a potent, possibly lethal immune response, generation of replication competent virus, and producing insertional mutagenesis due to random gene transfer that can lead to oncogenesis. Additionally, the capacity of viral vectors is restricted, which limits the size of genetic material that the vectors can accommodate. Nonviral vectors like liposomes offer promising alternatives for the delivery of DNA-based biopharmaceuticals [1–3] However, several problems still need to be addressed before liposomes can be used in a clinical setting. One of the major problems is poor stability and integrity of liposomes, which is partially attributed to the hydrolysis of ester bonds and oxidation of unsaturated fatty acids [4]. Tetraether lipids (TELs) are a unique class of lipids found exclusively in the cellular membranes of members of the third domain of life referred to as archaea, which can be considered promising alternatives to common lipids for liposomes intended for drug delivery. Members of the archaea are known to inhabit environments ranging from ordinary to those characterised by extremes of salt, temperature, and pH, for example, hypersaline and alkaline lakes, hot acid springs, and strictly anoxic settings [5–7]. The presence of ether lipids in membranes of the archaea is believed to be responsible for their unusual temperature and pH stability. Ether lipids differ considerably from prokaryotic and eukaryotic lipids, the main difference being the presence of hydrocarbon moieties linked to a glycerol/nonitol bridge group by an ether bond, which give the lipid molecule more resistance against hydrolytic damage than ester bonds in extremes of pH and temperature. Another difference is the structure of the hydrocarbons themselves; archaeal lipids consist of branched saturated isoprenoids, the most common being the C_20_ phytanyl, as opposed to straight chain unsaturated hydrocarbons in prokaryotic and eukaryotic membrane lipids. The lack of unsaturation protects ether lipids from oxidative damage [8]. Ether lipids can be broadly classified into two groups: monopolar and bipolar. Monopolar ether lipids (diether lipids) somewhat resemble common lipids in their structure, having two hydrocarbon chains linked by a bridge moiety to a single head group. Bipolar TELs are unique due to the presence of two head groups, one at each end of the molecule. They resemble two diether lipids whose tails have been covalently bound and are present in a monolayer in archaeal membranes. The monolayer arrangement is believed to add to the thermal and pH stability of the lipid membrane. TELs can be further subdivided into two subclasses, namely, glycerol-dialkyl-glycerol tetraether (GDGT) or glycerol-dialkyl-nonitol tetraether (GDNT) lipids. GDNT-based tetraethers constitute 70–80% or more of the total lipids of thermoacidophilic archaea such as* Sulfolobus acidocaldarius* [9, 10]. One of the promising applications of TEL liposomes is oral drug delivery owing to their stability in extreme pH conditions [10], as well as in the presence of serum, lipases, and bile salts. TEL liposomes have been proven to be a good carrier for the oral delivery of peptides like insulin [11], skin applications [12], and chlorine e6 for photodynamic therapy [13]. Tetraether lipids can be modified to obtain site specific interactions [14]. For gene delivery, synthetic or semisynthetic tetraether as well as diether lipids has been used [15–17]. In this study, native purified nonhydrolysed tetraether lipids (PLFE) and their hydrolysed backbones (hGDNT) from the archaeon* Sulfolobus acidocaldarius* were combined with the helper lipids cholesterol (CH), L-*α*-phosphatidylcholine (PC), and a cationic lipid 1,2-dioleoyl-3-trimethylammonium-propane (DOTAP) to prepare stable liposomes. Lipoplexes were then prepared by complexation with a luciferase expressing plasmid (pCMV-luc). The effectiveness of lipoplexes as gene delivery vehicles was investigated by performing in vitro transfection studies in SK-OV-3 cell line and compared against branched polyethyleneimine (25 kDa bPEI). Furthermore, the delivery vehicles were rated in relation to their ability to be used in oral gene delivery.

## 2. Materials and Methods

### 2.1. General Materials

Freeze-dried biomass of* Sulfolobus acidocaldarius* was obtained from SiT (Surface & Interface Technologies, Rosenhof GmbH, Heiligenstadt, Germany). N-[1-(2,3-Dioleoyloxy)propyl]-N,N,N-trimethylammonium chloride (DOTAP) was a gift from Lipoid (Ludwigshafen, Germany). L-*α*-Phosphatidylcholine was purchased from Avanti Polar Lipids (Alabama, USA) and cholesterol was obtained from Sigma-Aldrich (Taufkirchen, Germany). DNA intercalating dyes gel-red (10.000x in DMSO) and ethidium bromide (EtBr) were obtained from Invitrogen (California, USA) and Sigma-Aldrich (Missouri, USA), respectively. Purified plasmid pCMV-luc (pDNA) was purchased from Plasmid Factory (Bielefeld, Germany). Silica gel 60 (400–230 mesh) was obtained from Carl Roth GmbH (Karlsruhe, Germany). Reversed phase Chromabond C-18 and HPTLC (high performance thin layer chromatography) plates were bought from Macherey-Nagel (Weilmünster, Germany). Organic solvents chloroform (CHCl_3_), methanol (MeOH), and diethyl ether (DE) were obtained from VWR International (Pennsylvania, USA). Hydrochloric acid was purchased from Sigma-Aldrich. For 1H-NMR, deuterated chloroform (CDCl_3_) was obtained from Euriso-Top (Gif-sur-Yvette Cedex, France). All solvents used were of HPLC grade.

### 2.2. Cell Culture

SK-OV-3 adenocarcinoma ovarian cells were purchased from ATCC (Virginia, USA) and cultured in Iscove's Modified Dulbecco's Medium (IMDM), supplemented with 10% fetal calf serum. Cells were maintained as monolayers at 37°C with 5% CO_2_ and subcultured upon reaching 80% confluency. For transfection studies, Luciferase Cell Culture Lysis 5x Reagent and luciferin were purchased from Promega (Wisconsin, USA). Pierce BCA Assay was purchased from Thermo Scientific (Massachusetts, USA).

### 2.3. Isolation and Purification of Tetraether Lipids

#### 2.3.1. Polar Lipid Fraction E (PLFE)

6.3 g of freeze-dried biomass of* Sulfolobus acidocaldarius* was pestled for 15 min until a coarse fine yellow/greenish powder was obtained. It was then transferred into an extraction thimble for Soxhlet extraction. 500 mL mixture of CHCl_3_ : MeOH (50 : 50 v/v) was poured into a 1000 mL round bottom flask (RBF). Extraction was performed for 48 h using an oil bath at 80°C. After extraction, the organic solvent was evaporated leaving a crude lipid layer on the flask's wall. The crude lipid extract was then suspended in a small volume of MeOH : H_2_O (50 : 50 v/v) using an ultrasonic bath, Elmasonic P30 H from Elma Hans Schmidbauer, (Singen, Germany). The suspension was then transferred to a Chromabond C-18 column. The eluents used for the purification process were MeOH : H_2_O (50 : 50 v/v), CHCl_3_ : MeOH : H_2_O (22.5 : 55 : 22.5 v/v), and CHCl_3_ : MeOH : H_2_O (70 : 26 : 4 v/v).

#### 2.3.2. Hydrolysed Lipid (hGDNT)

0.5 g of freeze-dried biomass of* Sulfolobus acidocaldarius* was pestled and suspended in 4 M HCl in 500 mL RBF. The flask was put onto a reflux condenser for 24 h at 100°C. The suspension was filtered through a G-4 frit and the resulting filter cake was dried before being extracted five times with 50 mL chloroform at room temperature. The extract was concentrated and transferred onto a silica-gel column. Lipid material was purified by using the solvents CHCl_3_, CHCl_3_ : DE (80 : 20 v/v), and CHCl_3_ : MeOH (90 : 10 v/v). The last eluent was crucial in obtaining purified hGDNT.

### 2.4. Analysis of Tetraether Lipids

The purified hydrolysed lipids were analysed by mass spectrometry using a Q-Trap 2000 (Applied Biosystems, Foster City, USA) which operated at ion-spray ionisation (ESI-MS). The purified lipid hGDNT was diluted to 0.1 *μ*g/mL prior to measurement. IR spectrum of hGDNT was recorded by an ALPHA FT-IR spectrometer (Bruker Corp., Massachusetts, USA). 5 mg of hGDNT was used to obtain the transmission spectrum. For 1H-NMR studies, hydrolysed lipids were dissolved at 2.5 mg/mL in CDCl_3_ and analysed with a JEOL ECX-400 with an autotune sample head. HPTLC plates were used to identify the lipids PLFE and hGDNT using the mobile phases CHCl_3_ : MeOH : H_2_O (22.5 : 50 : 22.5 v/v) and CHCl_3_ : MeOH (90 : 10 v/v), respectively. Lipids were spotted using a MeOH/sulfuric acid spray reagent.

### 2.5. Preparation of Liposomes

Liposomes were prepared using the film hydration method [18]. Stock solutions of lipids were prepared by dissolving the lipids in a CHCl_3_ : MeOH (2 : 1 v/v) solution. Required amounts of lipid solutions were mixed in a 5 mL RBF and evaporated to dryness using a Laborota 400 rotary evaporator from Heidolph Instruments (Schwabach, Germany) creating a thin lipid layer on the wall of the flask (under vacuum, 280 rpm/min). The films were hydrated with 20 mM HBS buffer to obtain a lipid concentration of 6 mg/mL, and the flasks were placed in a bath sonicator and allowed to equilibrate for 5 minutes. Then, the flasks were sonicated for 2 minutes until the lipid film was fully reconstituted. Liposomal suspension was further extruded through 200 nm and 100 nm polycarbonate membranes (Whatman) using an Avanti Mini Extruder (Avanti Polar Lipids) to obtain small unilamellar liposomes. For cell-culture experiments, liposomes were filter-sterilised through a 0.22 *μ*m syringe filter and stored at 4°C up to two weeks.

### 2.6. Preparation of Lipoplexes

Liposomes and pDNA were diluted with OPTI-MEM. Equal volumes of pDNA solution and liposomal suspension were mixed and triturated several times to ensure homogeneous mixing. N/P ratio was calculated based on nitrogen containing DOTAP lipid (699 g/mol) and the phosphorus containing phosphate group for pDNA (330 g/mol). The mixture was allowed to stand for 20 minutes at room temperature (procedures were carried out under sterile conditions when preparing complexes for transfection studies).

### 2.7. Size and Zeta-Potential Measurements

Hydrodynamic diameter of liposomes and lipoplexes was determined by using Dynamic Light Scattering on a Malvern Zetasizer Nano ZS using Noninvasive Back Scatter (NIBS®). Liposomes were diluted (1 : 10) with Milli-Q water and equilibrated to 25°C prior to measurement. Measurement subruns were set automatically by the device depending upon the sample. Liposomes and DNA were complexed at N/P of 2.5 for 20 min before measurement. Zeta potential of liposomes and lipoplexes was determined at conductivity < 100 *μ*S/cm. The zeta potential was calculated using the Smoluchowski equation.

### 2.8. Atomic Force Microscopy (AFM)

Liposomes and lipoplexes were diluted (1 : 100) with Milli-Q water and pipetted onto a silicon wafer (1 × 1 cm^2^). After 10 min of incubation, suspension was removed by aspirating the excess of water leaving liposomes and lipoplexes on the silica wafer. Atomic force microscopy was performed on a NanoWizard® 3 NanoScience AFM from JPK Instruments (Berlin, Germany). The microscope was vibration-damped. Commercial 1-lever tips (NSC 14 Al/BS) on a cantilever with a length of 125 *μ*m and a resonance frequency of about 160 Hz and force constant of 5 N/m were used. All measurements were performed in tapping mode in air. Scan speed was adjusted between 0.5 and 1.5 Hz.

### 2.9. Cryo-Scanning Electron Microscopy (Cryo-SEM)

10 *μ*L of liposomal sample was placed onto a sample holder and immersed in liquid nitrogen. The sample was fractured with a fine blade in the preparation chamber of a JEOL JSM-7500F (Jeol Ltd., Tokyo, Japan) and sputter-coated with platinum to increase conductivity. The sample was then transferred into the SEM chamber. The instrument was maintained at −170°C all the time. Images were acquired at a voltage of 2.00 kV.

### 2.10. pDNA Intercalation Assay

150 *μ*L of lipoplexes at increasing N/P ratios was pipetted into an opaque 96-microtiter plate. Subsequently, 50 *μ*L of gel-red dye was pipetted into the complex suspension and fluorescence was immediately measured at the appropriate wavelength.

### 2.11. Toxicity Studies

*MTT*. Toxicity studies, for comparison of several lipoplexes and 25 kDa bPEI, were performed using the MTT (3-[4,5-dimethylthiazol-2-yl]-2,5-diphenyltetrazolium bromide) reagent (Sigma-Aldrich, Taufkirchen, Germany). 10.000 cells/well were seeded in 96-well plates and incubated overnight before addition of samples. After 24 h, the transfection reagents were aspirated and 200 *μ*L of 2 mg/mL MTT was added to each well, followed by further incubation of 3 h. Then, 200 *μ*L of DMSO was added to dissolve the formazan crystals. The formazan solution was analysed at 580 nm using a spectrometer. Triton X-100, PBS buffer (pH 7.4), and untreated cells were used as controls. Samples were analysed in triplicate.

*LDH*. LDH assay was performed using LDH Cytotoxicity Kit (Roche Diagnostics, Basel, Switzerland). Cells were seeded at a density of 10.000 cells/well and incubated overnight. On the following day, cells were treated with samples for 24 h. The supernatant was then transferred to a transparent 96-well plate and the LDH assay was performed according to the manufacturer's protocol. LDH activity was determined by monitoring the oxidation of pyruvate coupled with the reduction of NAD at 340 nm.

### 2.12. Transfection Studies

SK-OV-3 cells were seeded at a density at 10.000 cells/well 24 h prior to transfection. Lipoplexes were added to cells at different N/P ratios resulting in a pDNA amount of 0.5 *μ*g per well. Cells were incubated with lipoplexes for 4 h in IMDM medium to allow cellular uptake. The medium was then replaced by a fresh medium and the cells were further incubated for 48 h. Cells were subsequently washed with 200 *μ*L PBS buffer and 50 *μ*L of lysis buffer was added and placed in an orbital shaker for 30 min. Finally, 20 *μ*L of lysis buffer was transferred into an opaque 96-well microtiter plate. Expression of luciferase was measured by adding 50 *μ*L of luciferin to each well. Luminescence was expressed in RLU/mg (relative luminescence units/mg protein). The protein content was determined by pipetting 20 *μ*L of lysis buffer into a transparent 96-well plate. 200 *μ*L of bicinchoninic reagent was added and incubated for 30 min in an orbital shaker. In order to determine the protein content, the absorbance was measured at 562 nm and referenced against albumin standards.

### 2.13. Statistical Analysis

Data were expressed as mean ± SD from four individual samples. Statistical analysis was performed by using two-tailed Student's *t*-test in Microsoft Excel. Significant differences were represented by *p* < 0.05 (*∗*) and *p* < 0.01 (*∗∗*).

## 3. Results

### 3.1. Isolation, Purification, and Analysis of Tetraether Lipids

#### 3.1.1. Polar Lipid Fraction E (PLFE)

The high polarity of the first solvent MeOH : H_2_O (50 : 50 v/v) eluted mainly pigments, which fluoresced when exposed to UV light. The decreased polarity of the second eluent resulted in the PLFE. The final eluent CHCl_3_ : MeOH (90 : 10 v/v) gave less polar compounds. The fraction containing PLFE was analysed by HPTLC, where silica gel was the stationary phase and the eluent was composed of CHCl_3_ : MeOH : H_2_O (70 : 26 : 4 v/v). A spot at *R*_*f*_ 0.2 indicated the lipids (Figure 3).

#### 3.1.2. Hydrolysed Lipid (hGDNT)

The first solvent in the silica purification step CHCl_3_ eluted nonpolar substances like pigments (orange band). The second fraction which was eluted with CHCl_3_ : DE (80 : 20 v/v) gave the less unipolar substance hGDNT where no nonitol group is present. hGDNT was finally obtained in the last step using CHCl_3_ : MeOH (90 : 10 v/v) resulting in a dark brown band. hGDNT could be further purified by using cold acetone precipitation resulting in a resin-like appearance. The hydrolysed lipid hGDNT was analysed by HPTLC, where silica gel acted as a stationary phase and an eluent composed of CHCl_3_ : MeOH (90 : 10 v/v) was used. One single spot indicated hGDNT at *R*_*f*_ 0.2 (Figure 4).

Mass spectrometry (ESI-MS) of hGDNT resulted in a sharp signal at* m/z* 1479, which indicated the [M + Na]^+^ ion. Other characteristic signals are located at* m/z* 751 and 740, which represent [M + 2Na]^2+^ and [M + H + Na]^2+^ ions, respectively (Figure 5).

Recorded IR spectrum (Figure 5) indicates functional groups of hGDNT. A broad band at 3376 cm^−1^ corresponds to OH groups. Two sharp bands at 2921 cm^−1^ and 2854 cm^−1^ show CH, CH_2_, and CH_3_ groups. Bands at 1460 cm^−1^ and 1276 cm^−1^ are evident for CH2 and CH3, respectively. C-O-C (ether) and C-O-H (hydroxyl) groups are characterised by bands at 1102 cm^−1^ and 1085 cm^−1^, respectively. A sharp band at 758 cm^−1^ reveals C-H bend.

1H-NMR spectrum of hydrolysed GDNT is in accordance with that reported by Lo and Chang [18]: *δ* 0.7–0.9 (-CH_3_), 0.95–1.4 (-CH, -CH_2_), 1.45–1.9 (cyclopentyl -CH), 3.4–4.05 (-O-CH, -O-CH_2_) (Figure 6).

### 3.2. Size Determination

See Tables 1 and 2.

### 3.3. Atomic Force Microscopy (AFM)

Morphological characterisation of tetraether lipid containing liposomes using AFM showed mostly round shaped vesicles at sizes ranging between 50 nm and 100 nm which correlate with DLS measurements. Liposomes appeared in irregular shapes and spread onto the silicon surface resulting in a lipid monolayer (Figure 8). Lipid-pDNA complexes for all liposomal formulations showed different structures, resembling the often proposed “onion-like” structures (Figure 9). In contrast to liposomes, lipoplexes did not spread onto the silicon surface.

### 3.4. Cryo-Scanning Electron Microscopy (Cryo-SEM)

For liposome with tetraether lipids, only complete vesicles and no fracture planes were visible in Cryo-SEM, indicating the tight packaging of tetraether lipids within the lipid bilayer.

### 3.5. DNA Intercalation Assay

All liposomal formulations could complex pDNA in a concentration dependent manner until full complexation at N/P ratios of 2.5 to 3. This correlates perfectly with the highest transfection efficiency in this range. DOTAP and 25 kDa bPEI showed the most efficient complexation with pDNA, whereas all other liposomes were less effective in binding pDNA.

### 3.6. Toxicity Studies

See Figures 12 and 13.

### 3.7. Transfection Efficiency

Transfection studies with lipoplexes in SK-OV-3 cell line showed an overall reasonable transfection efficiency, which is comparable to the DOTAP standard. For binary mixtures, lipoplexes resulted in similar transfection efficiency to DOTAP. In contrast, incorporation of cholesterol and hGDNT into the bilayer resulted in an increase in transfection efficiency but decreased when PLFE was used as a stabilising agent. By incorporating PC and cholesterol, a 30% decrease in transfection efficiency was observed.

## 4. Discussion

**Figure 1:**
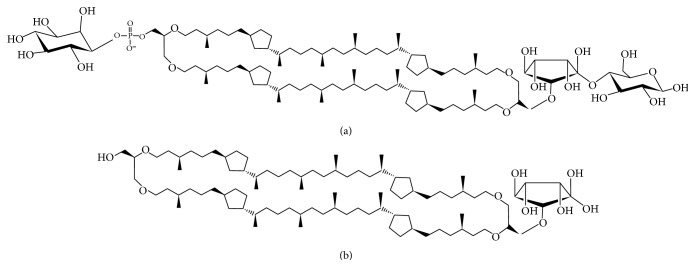
Structures of nonhydrolysed and hydrolysed fractions of tetraether lipids derived from* Sulfolobus acidocaldarius*. (a) PLFE and (b) hGDNT.

**Figure 2:**
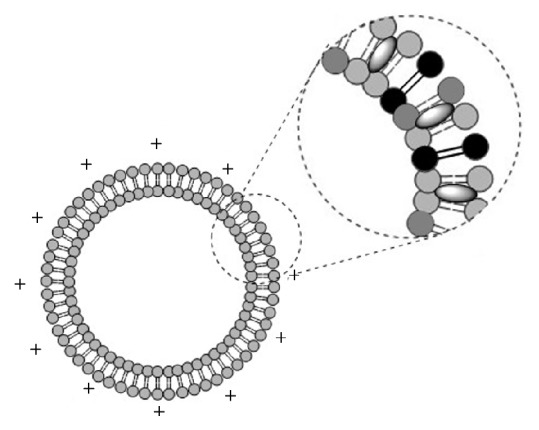
A model of a liposomal membrane containing tetraether lipids PLFE and hGDNT (black), complexing lipids DOTAP (dark grey colored), and helper lipids PC and CH (light grey, in between).

**Figure 3:**
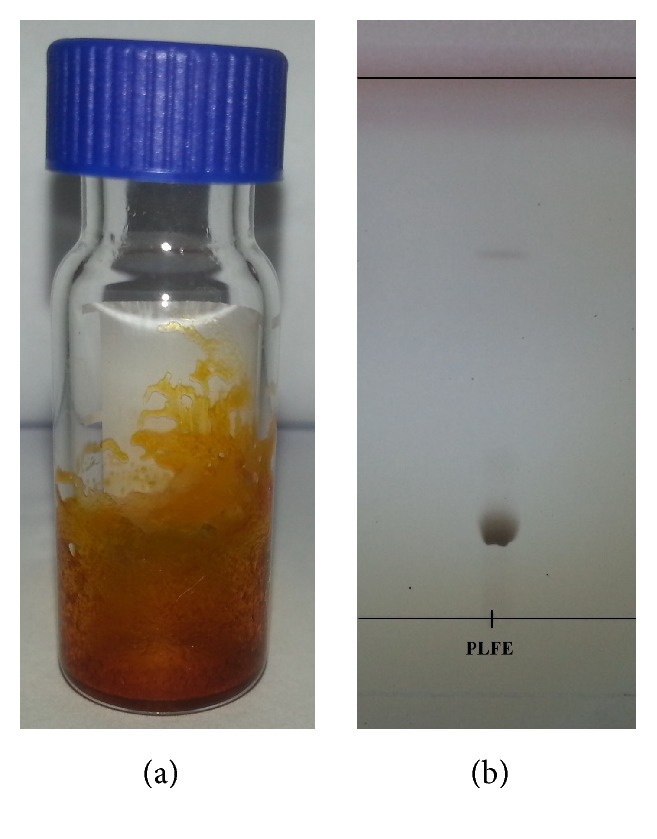
(a) Appearance of purified PLFE (19.6 mg). (b) HPTLC of PLFE with CHCl_3_ : MeOH : H_2_O (70 : 26 : 4 v/v) as a mobile phase and silica gel as a stationary phase.

**Figure 4:**
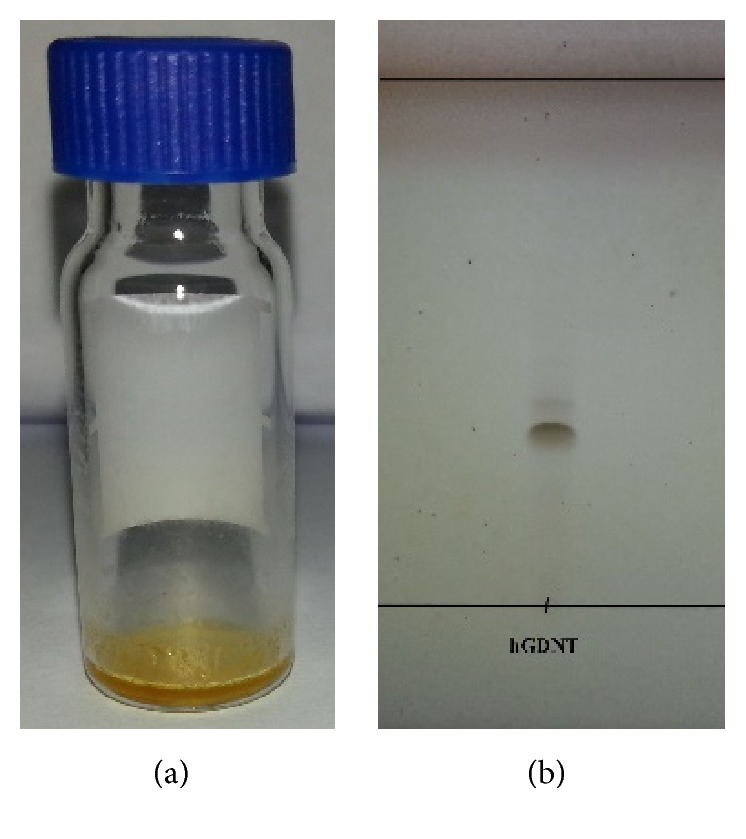
(a) Appearance of purified hGDNT (16.9 mg). (b) HPTLC of hGDNT with CHCl_3_ : MeOH (90 : 10 v/v) as a mobile phase and silica gel as a stationary phase.

**Figure 5:**
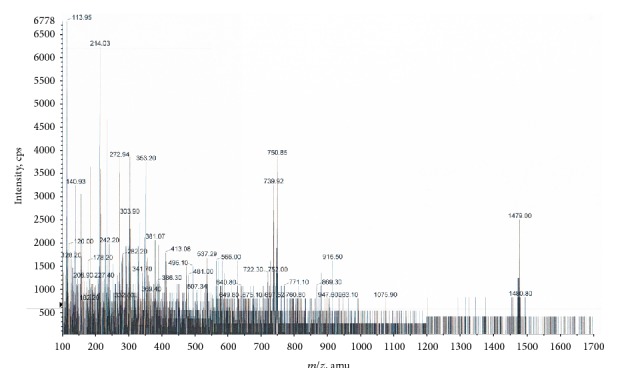
ESI-MS spectrum of purified hydrolysed lipid (hGDNT).

**Figure 6:**
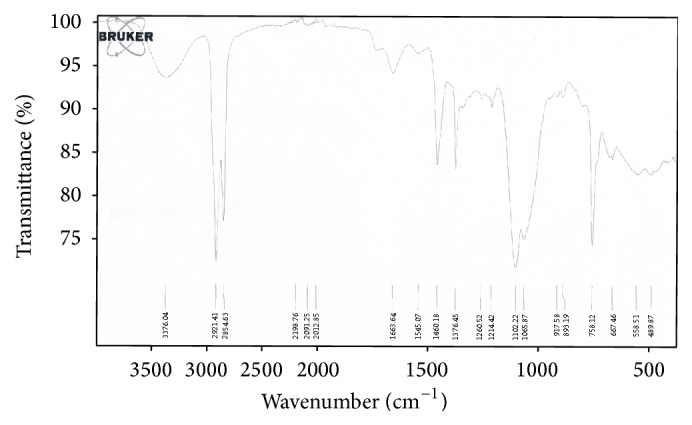
IR spectrum of hGDNT.

**Figure 7:**
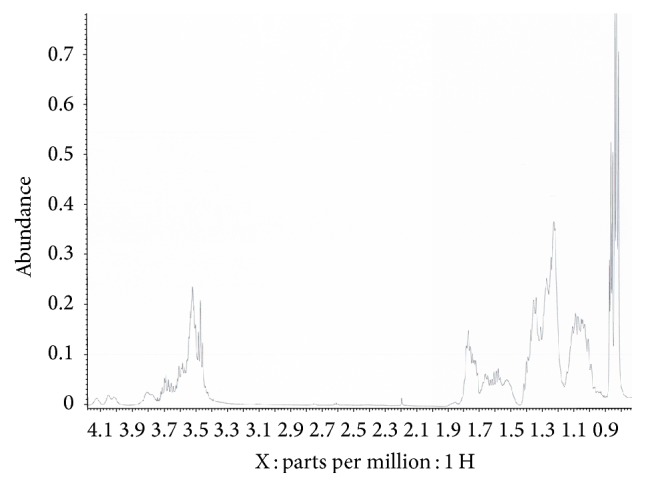
1H-NMR spectrum of hGDNT.

**Figure 8:**
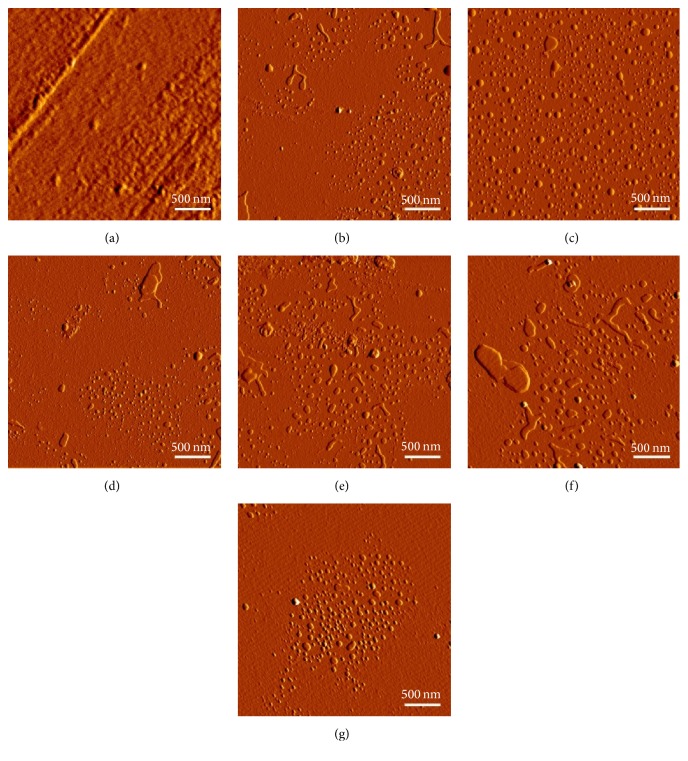
Visualisation of size and morphology of liposomes by atomic force microscopy. (a) 25 kDa bPEI, (b) DOTAP, (c) hGDNT/DOTAP (5 : 95), (d) PLFE/DOTAP (5 : 95), (e) hGDNT/CH/DOTAP (5 : 45 : 50), (f) PLFE/CH/DOTAP (5 : 45 : 50), and (g) hGDNT/PC/CH/DOTAP (5 : 30 : 35 : 30).

**Figure 9:**
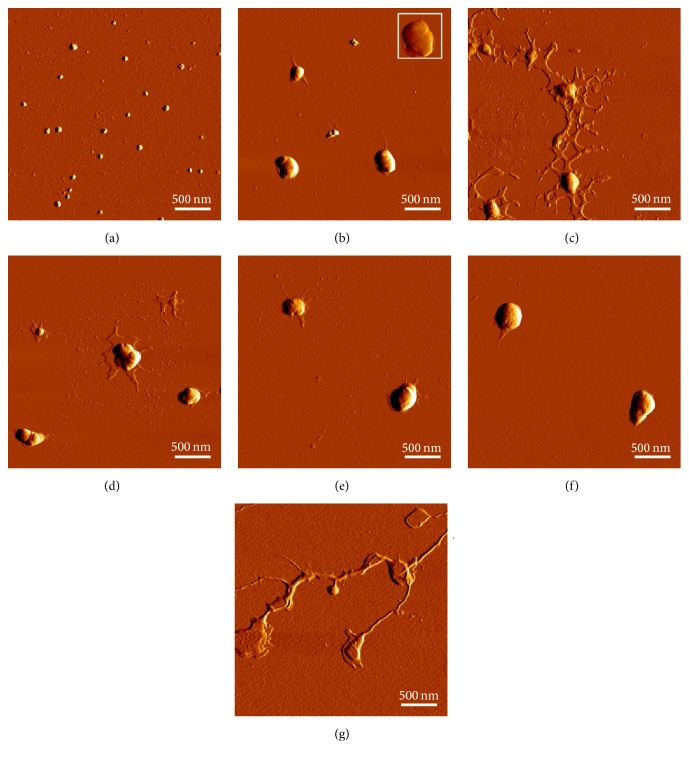
Visualisation of size and morphology of lipoplexes by atomic force microscopy. (a) 25 kDa bPEI, (b) DOTAP, (c) hGDNT/DOTAP (5 : 95), (d) PLFE/DOTAP (5 : 95), (e) hGDNT/CH/DOTAP (5 : 45 : 50), (f) PLFE/CH/DOTAP (5 : 45 : 50), and (g) hGDNT/PC/CH/DOTAP (5 : 30 : 35 : 30). All formulations were complexed at N/P 2.5–2.7. Inset: 500 × 500 nm.

**Figure 10:**
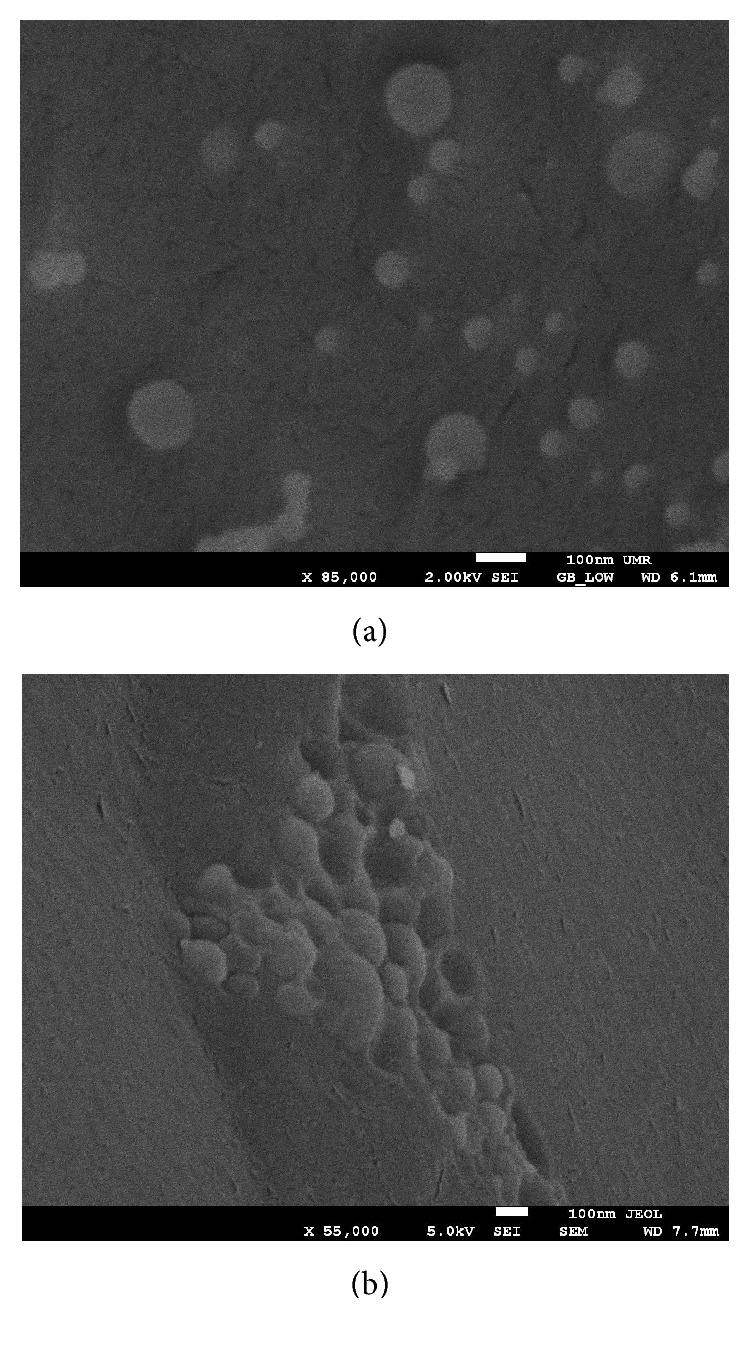
Cryo-SEM images of tetraether lipid containing liposomes. (a) hGDNT/DOTAP (5 : 95) and (b) hGDNT/PC/CH/DOTAP (5 : 30 : 35 : 30).

**Figure 11:**
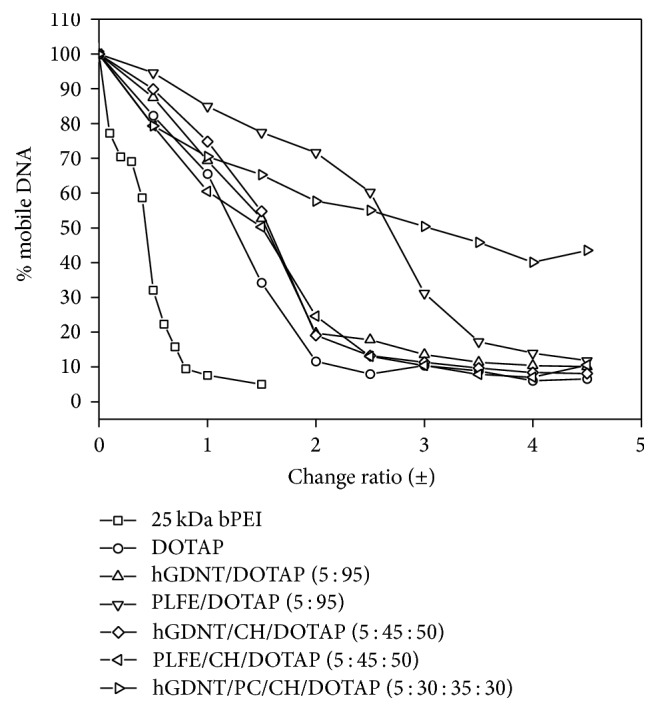
pDNA immobilisation of lipoplexes at increasing N/P (+/−) ratios. DNA complexes with increasing amounts of tetraether lipids (PLFE or hGDNT) were prepared and analysed with gel-red (1x).

**Figure 12:**
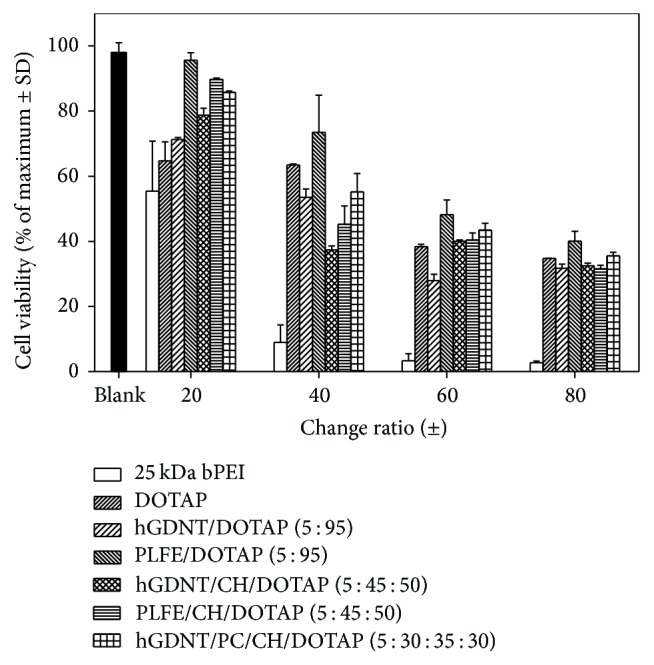
MTT assay of tetraether lipid containing lipoplexes and polyplexes.

**Figure 13:**
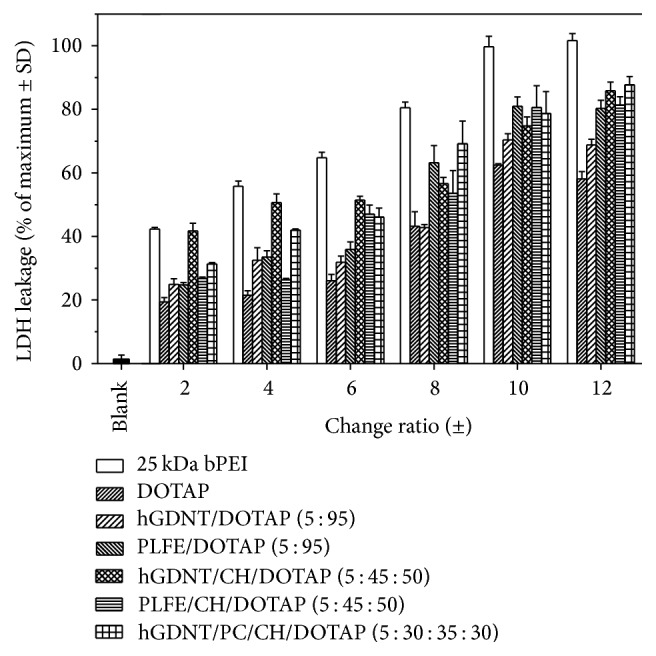
LDH assay of tetraether lipid containing lipoplexes and polyplexes.

**Figure 14:**
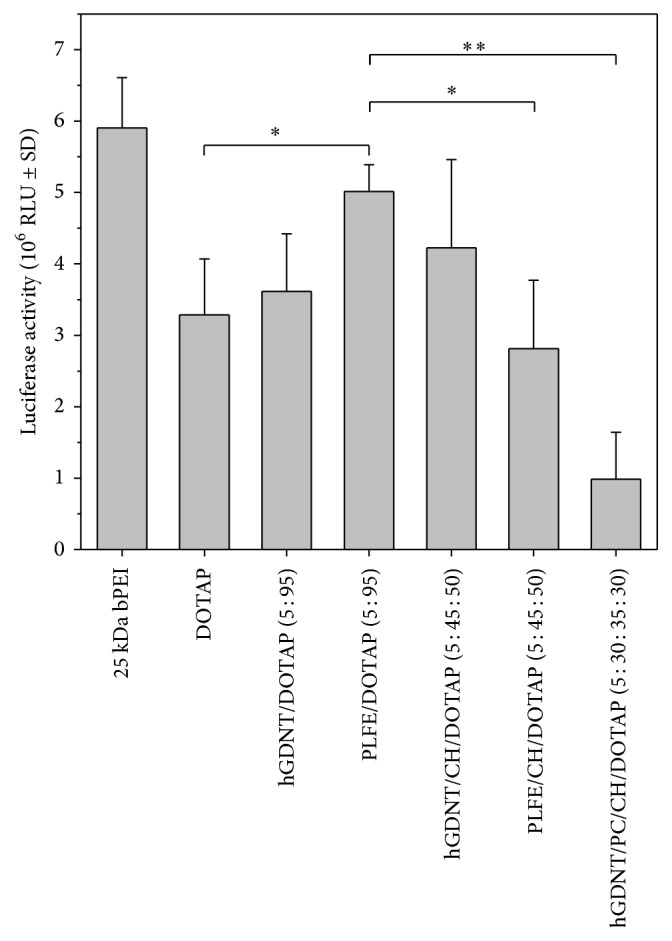
Transfection efficiency of various formulations. ^*∗*^*p* < 0.05; ^*∗∗*^*p* < 0.01.

To our knowledge, this is the first study which investigates the potential of native highly purified tetraether lipids, derived from* Sulfolobus acidocaldarius,* for the transfection of mammalian cells. A standardised and reproducible extraction process for two different fractions of tetraether lipids from S*ulfolobus acidocaldarius* was established. By applying Soxhlet extraction of the freeze-dried biomass, the sugar moieties as well as the phosphate group of the tetraether lipids remained intact (Figure 1(a)). Followed by purification on a Chromabond C-18 column, PLFE could only be eluted with a mobile phase composed of CHCl_3_ : MeOH : H_2_O (22.5 : 55 : 22.5 v/v), demonstrating the high polarity of sugar groups in PLFE (Figure 1(a)). HPTLC analysis with a highly polar mobile phase revealed *R*_*f*_ value of around 0.2 (Figure 3(b)), which is in accordance with Lo and Chang [18]. In contrast to the other findings, no further purification with methanol was necessary, because HPTLC analysis revealed a single spot for the PLFE fraction. Elferink et al. [19] had a range of spots in the corresponding fraction. Sugar moieties and the phosphate group could be removed by an acidic extraction process resulting in tetraether lipids with less polarity (Figures 1(b) and 4(a)). The procedure was more simplified in comparison to that of Bode et al. [20]. HPTLC analysis using a mobile phase of CHCl_3_ : MeOH (90 : 10 v/v) revealed lower polarity in comparison to PLFE. *R*_*f*_ value for hydrolysed lipids, which was around 0.3, was compared to the hGDNT standard obtained from Surface & Interface Technologies (Figure 4(b)). Further analysis of hydrolysed tetraether lipids by ESI-MS indicated a series of peaks around the theoretical mass (Figure 5). This is due to the presence of cyclopentane rings in the molecule. For the hydrolysed lipid, the sodium ions could be detected. The molecular weight measured by mass spectrometry is 23 g/mol above the theoretical masses, indicating 1.456 g/mol for hGDNT. 1H-NMR and IR spectrum of hGDNT (Figures 6 and 7) revealed relevant peaks and bands according to the analysis conducted by Parmentier et al. [21]. Preparation of liposomes, composed of tetraether lipids and DOTAP, was conducted at 5 mol% to take advantage of the stabilising effect of tetraether lipids, which are incorporated into the lipid bilayer (Figure 2). A similar observation was also made by Jensen et al. [22] where the highest fraction of tetraether lipid was 18 mol% in order to get stabilised liposomes. Furthermore, Parmentier et al. [23] stated that a molar fraction of 5–15 mol% hGDNT was sufficient in delivering sensitive peptides through the GIT. The morphology of liposomes, which was analysed by Cryo-SEM, revealed round shaped vesicles with no visible fracture planes, which is common for conventional liposomes (Figure 10). The absence of fracture planes can be explained by the tight membrane packaging of liposomes with tetraether lipids as stabilising agents, which was investigated by Khan and Chong [24]. AFM images of lipoplexes indicate “onion-like” structures (Figure 9(b), inset), where a liposome builds a core and pDNA is enwrapped around it. Another layer of lipids is then attached on it, followed by another layer of pDNA [25]. The complexation efficacy determined by using gel-red assay revealed successful complexation of liposomes with pDNA at N/P ratios around 2–2.5, where 80–90% of pDNA was complexed within the lipoplex (Figure 11). Superior complexation efficiency was observed in 25 kDa bPEI due to the polycationic nature of the polymer favouring particles with sizes of 60 nm (Table 2 and Figure 9(a)). Transfection efficiency for 25 kDa bPEI was high; however, the value is compared to liposomal DOTAP (Figure 14). This favours the usage of DOTAP over 25 kDa bPEI due to the lower toxicity in the MTT assay, where only 5% of cells survived at the highest N/P ratio for the polymer and 40% for DOTAP (Figure 12) indicating high loss in the metabolic activity of the cell. Moreover, toxicity of 25 kDa bPEI also affects the cell membrane resulting in higher leakage of lactate dehydrogenase (Figure 13) as seen in the LDH assay. The highest transfection efficiencies were obtained with lipoplexes with a positive zeta potential due to electrostatic effects that occur when lipoplexes interact with the negatively charged cell surface. A statistically significant transfection efficiency of PLFE/DOTAP (5 : 95) compared to DOTAP can be explained due to the stabilising effect of tetraether lipids, which prevents the denaturation of pDNA. A similar formulation hGDNT/DOTAP (5 : 95), where the sugar groups of the tetraether lipids have been removed, was less effective in delivering pDNA than PLFE/DOTAP (5 : 95). Considering almost equal diameters of both formulations of 214 nm and 322 nm, respectively (Table 2), as well as similar zeta potentials, the stabilising of the hydrolysed tetraether lipid was too high, resulting in stronger retention of pDNA. By incorporating the helper lipid cholesterol and lowering the fraction of DOTAP, for example, PLFE/CH/DOTAP (5 : 45 : 50) and hGDNT/CH/DOTAP (5 : 45 : 50), transfection efficiency was significantly lower than the superior liposomal formulation. Both lipoplex formulations with cholesterol revealed larger diameters (Table 2) compared to the other formulations. Despite charge, the size of a lipoplex is crucial in obtaining high transfection efficiencies. Lipoplexes, in the size range of 214 nm to 322 nm, showed the highest transfection efficiency due to the optimal surface, which promotes interaction with cell membrane. In the formulation comprising hGDNT/PC/CH/DOTAP (5 : 30 : 35 : 30), only 60% of pDNA could be complexed and protected (Figure 11). This confirms the high negative zeta potential of −48.6 mV (Table 1) indicating that the zeta potential is mainly determined by pDNA, which is enwrapped around the liposomes and in lipid tails with a diameter of around 10 nm (Figure 9(g)). Consequently, transfection efficiency was significantly lower compared to the other liposomal systems which was mainly due to the repulsive interaction of negatively charged lipoplexes with negative cell surface. This is in agreement with experiments from Tabatt et al. [26] who investigated novel lipid-DNA vectors, which had a negative surface charge. In creating a negatively charged lipoplex for gene delivery, thrombocyte or bile salt aggregation can be avoided when bringing the vehicle into the human body by injection or via the oral route [27]. Moreover, negatively charged particles can be used for the pulmonal delivery of genetic material without interacting with the mucous gel containing highly glycosylated segments.

## 5. Conclusion

Various tetraether lipids could be extracted, purified, and incorporated into spherical bilayers up to a certain amount resulting in liposomes that are nanoscaled and stable. Transfection agents based on tetraether lipids could be easily prepared by adding the cationic lipid DOTAP and pDNA resulting in stable and positively charged lipoplexes. In this study, we have shown that lipoplexes containing tetraether lipids have a statistically significant higher transfection efficiency compared to DOTAP alone. Moreover, a formulation was identified that has the potential to be used for the oral gene therapy by circumventing platelet and bile salt aggregation.

## Figures and Tables

**Table 1:** Composition and physicochemical properties of polymer and liposomal formulations.

Formulation (mol : mol)	Particle size ± SD [nm]	AFM diameter [nm]	Zeta potential [mV]
25 kDa bPEI	—	—	—
DOTAP	84.7 ± 3.4	65.3	+38.5
hGDNT/DOTAP (5 : 95)	89.8 ± 6.9	67.5	+40.9
PLFE/DOTAP (5 : 95)	94.3 ± 6.9	70.8	+38.1
hGDNT/CH/DOTAP (5 : 45 : 50)	96.6 ± 8.8	63.6	+41.1
PLFE/CH/DOTAP (5 : 45 : 50)	103.7 ± 49.4	71.3	+45.5
hGDNT/PC/CH/DOTAP (5 : 30 : 35 : 30)	96.6 ± 8.8	67.9	+ 48.6

**Table 2:** Composition and physicochemical properties of polyplexes and lipoplexes.

Formulation (mol : mol) [N/P ratio]	Particle size ± SD [nm]	AFM diameter [nm]	Zeta potential [mV]
25 kDa bPEI [5.0]	60.2 ± 10.2	79.5	+31.2
DOTAP [2.5]	220.1 ± 26.8	257.1	+38.9
hGDNT/DOTAP (5 : 95) [2.5]	322.1 ± 49.9	317.4	+41.0
PLFE/DOTAP (5 : 95) [2.5]	214.5 ± 10.2	210.7	+42.7
hGDNT/CH/DOTAP (5 : 45 : 50) [2.7]	450.1 ± 4.5	473.5	+50.1
PLFE/CH/DOTAP (5 : 45 : 50) [2.7]	372.3 ± 15.0	441.3	+51.6
hGDNT/PC/CH/DOTAP (5 : 30 : 35 : 30) [2.7]	231.8 ± 35.0	398.4	−48.6
